# Analysis of differential β variable region of T cell receptor expression and NAV3/TNFRSF1B gene mutation in mycosis fungoides

**DOI:** 10.18632/oncotarget.7673

**Published:** 2016-02-24

**Authors:** Hongzhou Cui, Jie Liu, Li Li, Jingyu Ren, Shuping Guo, Li Bai

**Affiliations:** ^1^ Department of Dermatology, The First Hospital, Shanxi Medical University, Taiyuan, Shanxi 030001, PR China; ^2^ Jiangxi Provincial Center for Disease Control and Prevention, Nanchang, Jiangxi 330029, PR China

**Keywords:** mycosis fungoides, T cell receptor beta, NAV3, TNFRSF1B

## Abstract

**Objective:**

This study aimed to analyze the predominant expression of the variable region of T cell receptor (*TRBV*) and determine whether *NAV3* or *TNFRSF1B* gene mutation involved in the pathogenesis of MF.

**Results:**

*TRBV5-7* expression increased from the normal, early-stage to advanced-stage lesion in MF patient. By contrast, *TRBV2* decreased with the lesion developed. We found no mutations of *NAV3* or *TNFRSF1B* in the lesions from this study.

**Materials and Methods:**

Real-time PCR were used to screen differential expression of *TRBV* in different lesions. Mutational analyses were used to validate genetic alterations in the skin lesions.

**Conclusions:**

The identification of *TRBV* gene expression differences between normal and different stages of MF lesions provide insight into promising new diagnostic and prognostic biomarkers. None of the reported genetic abnormalities suggests complexity of progress from a primary cytogenetic event to an advanced stage with poor prognosis in MF.

## INTRODUCTION

Mycosis fungoides (MF) is a malignant T-cell lymphoma of the skin, which was firstly reported by Alibert in 1806. The incidence rate is accounted to 1 in 300,000 around the world. MF is a chronic disease with indolent course range from years to decades [1]. It is clinically divided into 3 stages according to morphological feature. The natural history usually begins with flat patches (patch stage), small to medium cerebriform nuclei lymphocyte colonize the basal layer. Over years, the infiltrated lesions becomes more marked and leads to extensive plaques (plaque stage), which featured as pronounced epidermotropism and pautrier microabscesses. In tumor stage, various sized and shaped nodules developed on infiltrated plaques, painful superficial ulceration widespread and superficial ulceration occur eventually. The lymphocytes with cerebriform nuclei mainly infiltrated in the dermis and subcutaneous fat, while the epidermotropism are not apparent.

T-cell receptor (TCR) signaling is considered as an essential component in lymphomagenesis. The constitution of CDR3 by *TRBV* gene rearrangement has been reported a useful indicator in the diagnosis and monitoring of MF [2]. However, few studies on differential expression of *TRBV* had been performed among skin tissue in different stages. Recently, the genetic factors had been thought to be involved in the pathogenesis of MF. Karenko et al. revealed *NAV3* deletion in skin lesions samples was carried by 50% of the early MF and 85% of the advanced MF and Sezary's syndrome (SS) [3]. Additionally, recurrent *TNFRSF1B* point mutations were also reported to contribute to 18% of patients with MF [4].

Here, we reported a patient diagnosed with MF that presents a typical patch, plaque and tumor on trunks and extremities. *TRBV* differential expression was performed among different stage lesions in MF. We also determine whether mutated *NAV3/TNFRSF1B* gene involved in the pathogenesis of MF.

## RESULT

The result showed that Ct values of *TRBV5-7* and *TRBV12* were increased in the MF patient compared with healthy subjects. In addition, the expression of *TRBV5-7* revealed a upward trend from normal phenotype, early-stage to advanced-stage lesion in MF patients. *TRBV2* showed a decreased expression with the lesion developed. However, *TRBV24* presented a differential expression upon different lesions, but no trend was found among them (Table 1).

**Table 1:** ΔCT of *TRBV* genes in different lesions of MF and healthy subject s

	Patch-plaque stage lesion	Tumor stage lesion	Self normal lesion	Health lesion
*TRBV2*	6.12	4.85	7.97	3.21
*TRBV5-7*	4.23	6.97	2.89	2.45
TRB12	6.32	6.00	3.28	3.79
*TRBV24*	3.18	7.99	4.21	4.35

Note: ΔCT = differential threshold cycle value between *TRBV* and TRAC.

Mutational analysis identified no mutations of *NAV3* or *TNFRSF1B* in the different stages of MF lesions. However, we could not exclude the potential existence of other mutations that may be involved in the pathogenesis of MF.

## DISCUSSION

MF is a primary epidermotropic T-cell lymphomas (TCL) characterized by progression through patches, plaques and tumor stage disease. This case constituted a typical clinical evolution. Besides of simultaneous supporting lesions and lymph node involvement, a combination of pathological, immunological and laboratory test reaches a conclusive diagnosis of MF.

TCR gene rearrangement studies can be highly useful in the diagnosis and monitoring of MF. It is believed that T cells express either αβ or γδ TCR chains. The development of αβ lineage cells requires the expression of a pre-TCR α chain, which associates with a functional TCR-β chain to form a pre-TCR. Only thymocytes that express a functional pre-TCR can efficiently mature to become final single CD4 or CD8 mature αβ T cells.

The constitution of CDR3 by *TRBV* gene rearrangement is essential for T cell antigen recognition [5]. Analysis of CDR3 polymorphism and length has been used to monitor proliferation of tumor T cells and specific responsive T cells. Since MF is a T cell-originated skin disease, it is reasonable to believe that T cells with specific *TRBV* CDR3 spectral types can invade skin tissue after activating at the primary stage of MF. The persistent stimulation of these T cells may lead to a malignant clone development and increase the number of circulation malignant T-cells at the advanced stage [6]. Therefore, analysis of *TRBV* CDR3 polymorphism can reveal the mechanism by which T cells stimulate the onset and development in MF at the molecular level. In this study, we observed a selective expression of *TRBV* among different stages of MF. The differential expressed *TRBV* gene may contain the functional sites of these specifically activated T cells. Furthermore, additional studies are warranted to replicate and extend these findings in more MF patients.

The genetic basis of MF remains incompletely characterized. Several studies had revealed the loss of the *NAV3* signals is the most recurrent abnormality in the pathogenesis of MF [3, 7]. However, following studies did not support a consistent *NAV3* gene variation pattern, only a small proportion of the MF/SS are involved in 12q deletion including *NAV3* [8]. More recently, Ungewickell et al. reported 18% of patients with MF/SS carrying mutations in *TNFRSF1B*. In addition, other chromosomal alterations, genomic gains and losses, and differences in miRNA expression were also identified to associate with these malignancies [4]. These results suggested an underlying genomic instability in MF. The identification of genetic instability would help to identify patients with MF who progress to an advanced stage with poor prognosis.

We also assessed the *NAV3*/*TNFRSF1B* genes, but did not identify any potential mutations in MF. These and previous results suggest that MF is a genetically heterogeneous disorder, and it is likely that more than one genetic locus is responsible for the development of this peculiar disease. Further study, using extensive sequencing of noncoding or regulatory sequence of these genes is warranted.

## MATERIALS AND METHODS

### Patient

**Figure 1:**
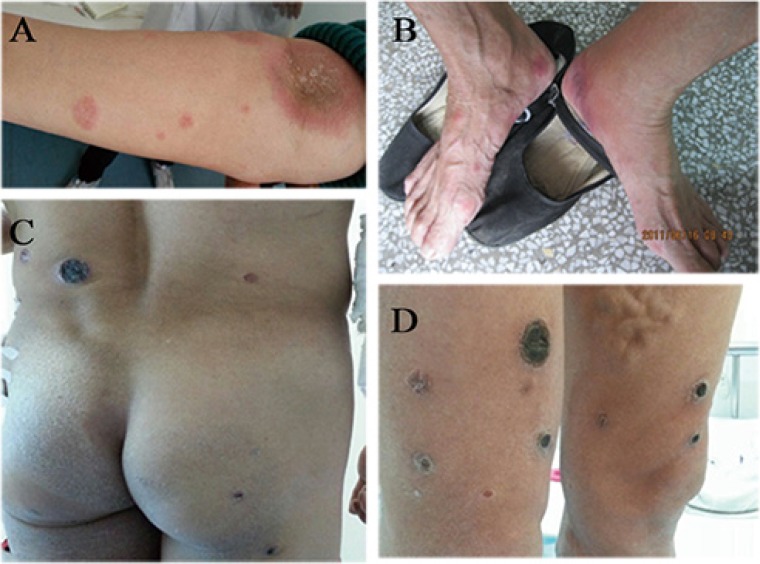
Clinical features in a patient with MF (**A**) Infiltrated plaques on the right arms. (**B**) red patches on the feet. (**C**–**D**) ulcerations and dark crusts on the back and legs.

A 54 year-old Chinese man was presented to First Hospital of Shanxi Medical University with diffuse red macule, plaque and ulcer and erosion with feeling of itch and ache. Red papules started 6 years ago. The lesions subsided after 2–3 months and relapsed on extremities in the following 2 years. Papules-plaques lesions were more infiltrated and subcutaneous palpable nodules present in some of the lesions on extremities, erosions occurred; Then, subsequently and progressed to dark crusts. The patient had a 2 years history of unprovoked baldness in occiput. He denied a family history of skin condition. Dermatologic examination revealed discoid patches or plaques on extremities varying in size from 2–8 cm, some of lesions have nontender and firm nodules on infiltrated plaques. The lower abdomen, buttocks and thighs were involved with deep oval ulcers; while the firm bases covered with a necrotic grayish substance and rolled edges. He had a remarkable alopecia areata about the size of the palm of a hand in occiput, with palpable nodule under the skin. Physical examination showed a enlarged axillary and inguinal lymph nodes (Figure 1).

Laboratory tests were revealed as follows: White blood cell: 9.4 × 10^9^/L (4.0–10.0 × 10^9^/L), Red blood cell: 4.0 × 10^12^/L (3.5–5.5 × 10^12^/L), Hemoglobin: 104 (110–160 g/L), Neutrophils%: 63.9% (30–70%), Lymphocytes: 31.2% (20–40%), Serum creatinine: 128.9 umol/L (35–97 umol/L), Erythrocyte sedimentation rate: 35 mm/h (0–15 mm/h).

Bone marrow suggested erythrocytosis, guanulocytosis thrombocytosis. The rate of heterotypic lymphocytes were significantly increased.

**Figure 2:**
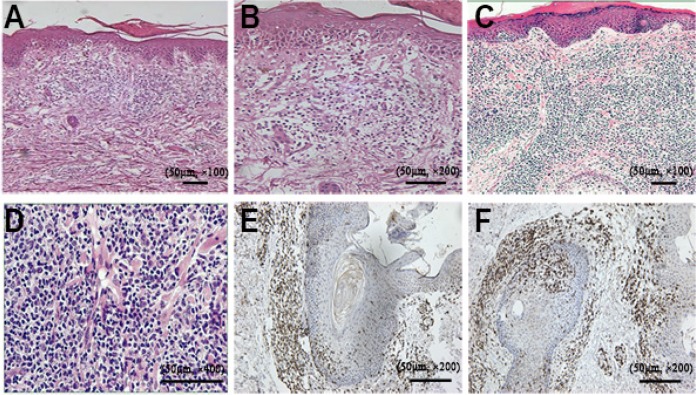
Pathological features in the reported case (**A**) H & E staining (× 100) showing the lymphocytes distributing in superficial perivascular and variably interstitial, a few lymphocytes enter into the epidermis. (**B**) higher-power view of the image (200 ×) showing lymphocytes infiltrated into the epidermis. (**C**) H & E staining (× 100) showing dense infiltrating lymphocytes in the dermis and subcutaneous fat. (**D**) higher resolution (× 400) highlighting the atypical lymphocytes with more pleomorphism. Immunohistochemical staining showing CD4^+^ (**E**), CD8^+^ (**F**) expressed in MF neoplastic cells.

Histological examination taken from patch-plaques lesions showed a band-like lymphoid infiltration, epidermotropism, slight prominent spongiosis, dermal fibrosis and follicular mucinosis. In tumor lesions, dense sheets of lymphocytes infiltrated in the dermis and subcutaneous fat, We also observed a moderate heterotypical change of the lymphocytes (Figure 2).

Immunohistochemistry tests showed CD3, CD4, CD8, CD20, PAX-5 positively expressed. Chest x-ray, computed tomography (CT) and ultrasonography captured no visceral tumors (Figure 2).

Combined of the tests and modified ISCL/EORTC revisions of MF, the case can be diagnosed with MF into stage IIB (T3N1M0B0) [9]. Informed consent was obtained from the participant. This study was authorized by the Ethics Committee of Shanxi Medical University and was conducted in accordance with the principles of the Declaration of Helsinki.

### Experimental research

Genomic DNA & RNA were extracted from biopsy specimens, which obtained from the unaffected, patch-plaque and tumor skin lesion and a normal prepuce tissue using DNA & RNA isolation reagents (Takara Biotechnology, Dalian, China). RNA purification was performed according to the manufacturer's protocol; cDNA was synthesized by AMV reverse transcriptase using an oligo dT primer (Promega, Madison, WI).

*TRBV* specific primers were chosen from published studies (Yin GH, et al. Eur J Dermatol 2011; 21(6): 938–44), and primers with the highest efficiency were used in this study. A sequence within T cell receptor β chain constant region (*TRBC*) was used for the common reverse primers [10]. Following primers from T cell receptor α chain constant region (TRAC) were used as the internal control: 5′-gcatgtgcaaacgccttcaacaacagc-3′ and 5′-cgggtttaatctgctcatgacgctgcggct-3′.

**Figure 3:**
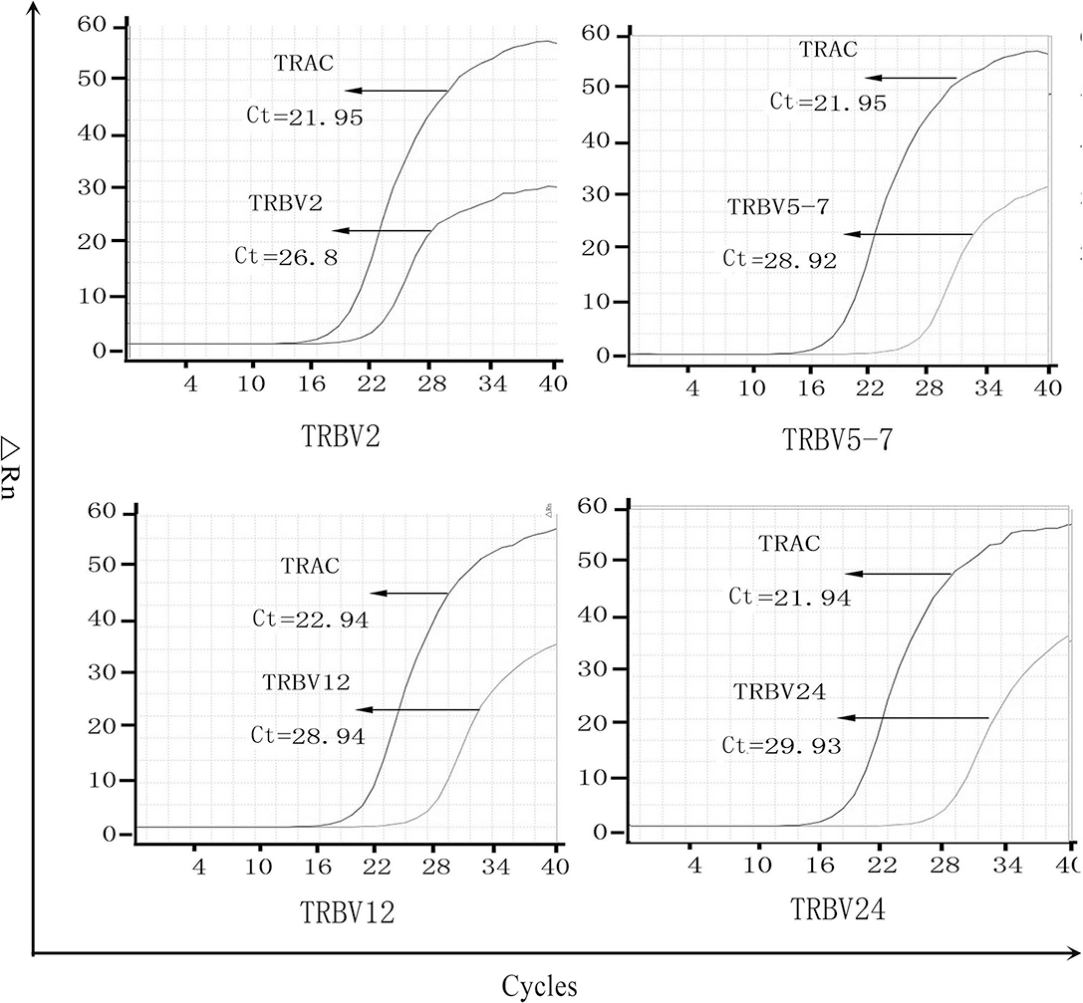
The PCR amplification curve of differential expression of *TRBV* in tumor lesions TRAC is used as the internal control, lower Ct represents higher expression, and the real expression can be standardized by ΔCT.

The fluorescent quantitative PCR (FQ-PCR) reactions were performed in StepOne real-time PCR apparatus (Applied Biosystems, Carlsbad, USA) using an SYBR Premix EX Taq 11 (Perfect Real Time) (Takara Biotechnology, Dalian, China). As described in Marion Marty's study, the relative expression level was obtained by subtracting the threshold cycle values (Ct) of TRAC from *TRBV* (Figure 3) [8].

We also determined the *NAV3* and *TNFRSF1B* gene expression in MF lesion. Genomic DNA from the unaffected, patch-plaque and tumor lesions were extracted using a standard phenol-chloroform protocol. Primes cover all the exons and intron-exon boundaries of candidate genes were designed using the Primer Premier 5.0 program. PCR amplification were performed by ABI 9700 Thermal Cycler (Applied Biosystems, Carlsbad, CA). The products were purified using QIAquick PCR Purification Kit (Qiagen, California, USA) and sequenced using the ABI PRISM 3730 automated sequencer (Applied Biosystems, Carlsbad, CA). Chroma (V3.0) was used to read the amplification fragment.
